# Introduction of a modified Degan classification to specify treatment algorithms in fractures of the anterior process of the calcaneus

**DOI:** 10.1186/s12891-022-05903-8

**Published:** 2022-10-28

**Authors:** Charlotte Cibura, Raimund Lülsdorff, Tim Ramczykowski, Thomas Armin Schildhauer, Christiane Kruppa

**Affiliations:** Department of General and Trauma Surgery, BG-University Hospital Bergmannsheil Bochum, Ruhr-University Bochum, Bürkle-de-la-Camp-Platz 1, 44789 Bochum, Germany

**Keywords:** Anterior process of the calcaneus, APC fracture, Degan classification, Calcaneocuboid joint

## Abstract

**Background:**

Fractures of the anterior process of the calcaneus are often missed, and their treatments and results receive little attention in the current literature. The aim of this study was to specify treatment algorithms through a modification of the Degan classification.

**Methods:**

Between 2009 and 2019, patients with APC fractures were retrospectively analyzed. The Degan classification was used and modified. Type III fractures were further divided into subgroups A (not displaced) and B (displaced). The type of treatment and complications were recorded. Return to work and posttraumatic osteoarthritis were determined as primary and secondary outcome parameters, respectively.

**Results:**

Forty-one patients with 43 fractures were included. Follow-up averaged 35,5 months (range 1,5–152 months). Fractures were eight type I, six type II, 15 type IIIA and 14 type IIIB. The fracture was initially recognized in 29 (70,7%) patients, and missed in 12 (29,3%) patients, respectively. Overall, the delayed diagnosed fractures had a significantly higher complication rate (*p* < 0,000) than the initially diagnosed fractures and received surgical treatment significantly (*p* < 0,009) more often.

After surgical treatment of 13 type IIIB, one nonunion occurred. Six missed type IIIA fractures were treated surgically after delayed diagnosis because of persistent symptoms. Two type I fractures required arthrodesis of the Chopart joint. Four patients did not return to work during the follow-up (3 missed type IIIA fractures, 1 type II fracture).

**Conclusion:**

Missed APC type IIIA fractures are at risk to develop complications, which is why computed tomography diagnostics should be performed if there is any clinical suspicion.

## Background

Fracture of the anterior process of the calcaneus (APC) has been described as a rare fracture [1–3]. However, due to limited radiological capabilities, many injuries might have been missed in the past [4]. Since the increasing use of computed tomography (CT) and magnetic resonance imaging (MRI), APC injuries have accounted for up to 38% of all extraarticular calcaneus fractures and are often associated with other injuries at the Chopart joint line [5–7]. In the few studies to date, which are predominantly case reports, various surgical and conservative treatment options have been described, but mostly with low case numbers and often without a uniform classification or consensus on therapy [1, 2, 5, 8–17]. In addition, APC fractures can only poorly be classified using the current fracture classifications of the calcaneus [2, 18–20]. The Degan classification, introduced in 1982, has mainly been used for these factures, and no uniform therapy recommendations based on the classification exist [5]. The Degan classification differentiates a nondisplaced avulsion fracture without the involvement of the calcaneocuboidal (CC) joint (type I), a displaced fracture without the involvement of the CC joint (type II) and a displaced fracture with the involvement of the CC joint (type III) on the basis of a lateral X-ray image.

However, the classification does not include nondisplaced fractures with the involvement of the articular surface, which are often only detected by CT diagnostics. Overall, there is a lack of more recent studies with high case numbers and a uniform extended classification based on today’s diagnostic possibilities.

The aim of this study was therefore to classify and evaluate APC fractures using sagittal CT. For this purpose, the Degan classification was modified and used, and treatment outcomes were differentiated among the fracture types, including dislocated and nondisplaced fractures with the involvement of the CC joint, to specify treatment recommendations based on the results. The main hypothesis was that the Degan classification Type III fractures in fact should be subdivided into two separate groups (fractures without intraarticular dislocation and those with intraarticular dislocation) demanding different treatment algorithms (nonoperatively versus operative treatment). Thus, we proposed the mentioned modification to the classification. Furthermore, we hypothesized that complicating factors such as delayed time of diagnosis significantly influence clinical outcome and the amount of treatment required.

## Methods

The present study was performed in accordance with the Declaration of Helsinki and its later amendments. Ethical permission for this study was obtained from the ethics committee (registration number: 20–6865-§23b).

### Study design

This was a retrospective register study over a period of 10 years in a level 1 trauma center. All patients with an injury to the APC treated in our hospital from 01/2009 until 12/2019 were included. Patients with injuries that were initially missed and delayed treated were also included but were considered separately. The exclusion criteria were as follows:

1. Additional tongue-type or joint depression fractures of the calcaneus
2. Lack of accurate diagnostics, such as CT or MRI
3. Follow-up of less than 6 weeks
4. Age < 18 years

To capture all patients with these criteria, a keyword analysis of all digitized files was performed by the authors. The key words were “anterior calcaneal process fracture” and “avulsion fracture”; in addition, all fractures coded as calcaneus fractures were checked for isolated injuries to the APC. The medical records of these patients were reviewed for the following factors: age, sex, trauma mechanism, concomitant injuries, fracture treatment (nonoperative vs. operative, tip toe weight bearing vs. full weight bearing) and complications such as the necessity of operative revisions and nonunion. The primary outcome parameter was defined as return to work, and the development of posttraumatic osteoarthritis was evaluated as a secondary parameter.

In the abovementioned period, a total of 50 patients with a fracture of the APC were found; however, nine patients had no documented follow-up of at least 6 weeks and were excluded. Thus, 41 patients with 43 fractures (follow-up [FU] rate 82%, *n* = 41) were included in the study.

The data were collected anonymously using Microsoft Excel© Version 14.7.7. Statistical analysis was performed using IBM SPSS Statistics 27. Hypotheses were tested using a Pearson chi-square test. The significance threshold was defined as 0.05.

Patients were subdivided according to initially diagnosed fractures of the APC and missed APC injuries. Operative and nonoperative treatment methods were differentiated in both groups and in the case of additional Chopart injuries or additional lower extremity injuries, these were listed separately.

### Modified classification

**Fig. 1:**
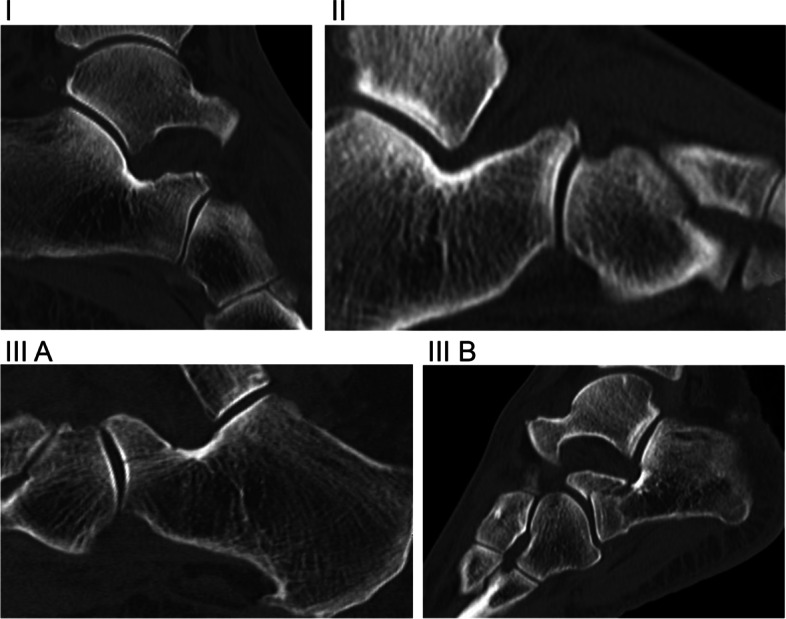
Type I - Nondisplaced fracture of the apex of the APC. Type II - Displaced fracture that does not include the articular surface. Type III A - Large fragment, intraarticularly, not displaced without joint step. Type III B - Large fragment, intraarticularly, displaced with joint step

**Fig. 2:**
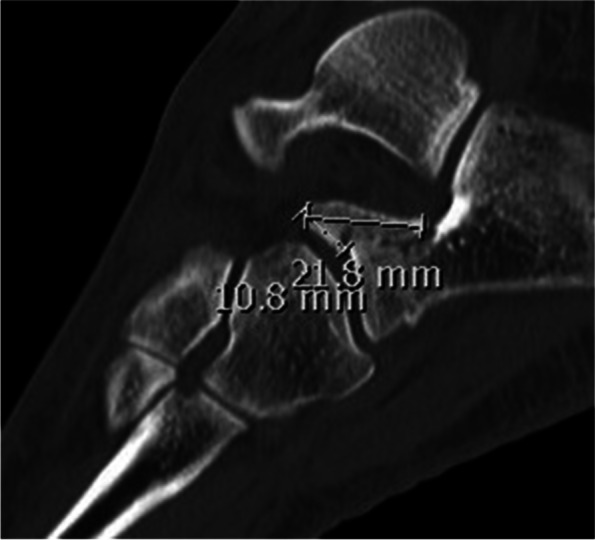
Measuring the fragment size in the sagittal CT with an estimated digital measurement tool (Impax, Agfa, Germany)

**Fig. 3:**
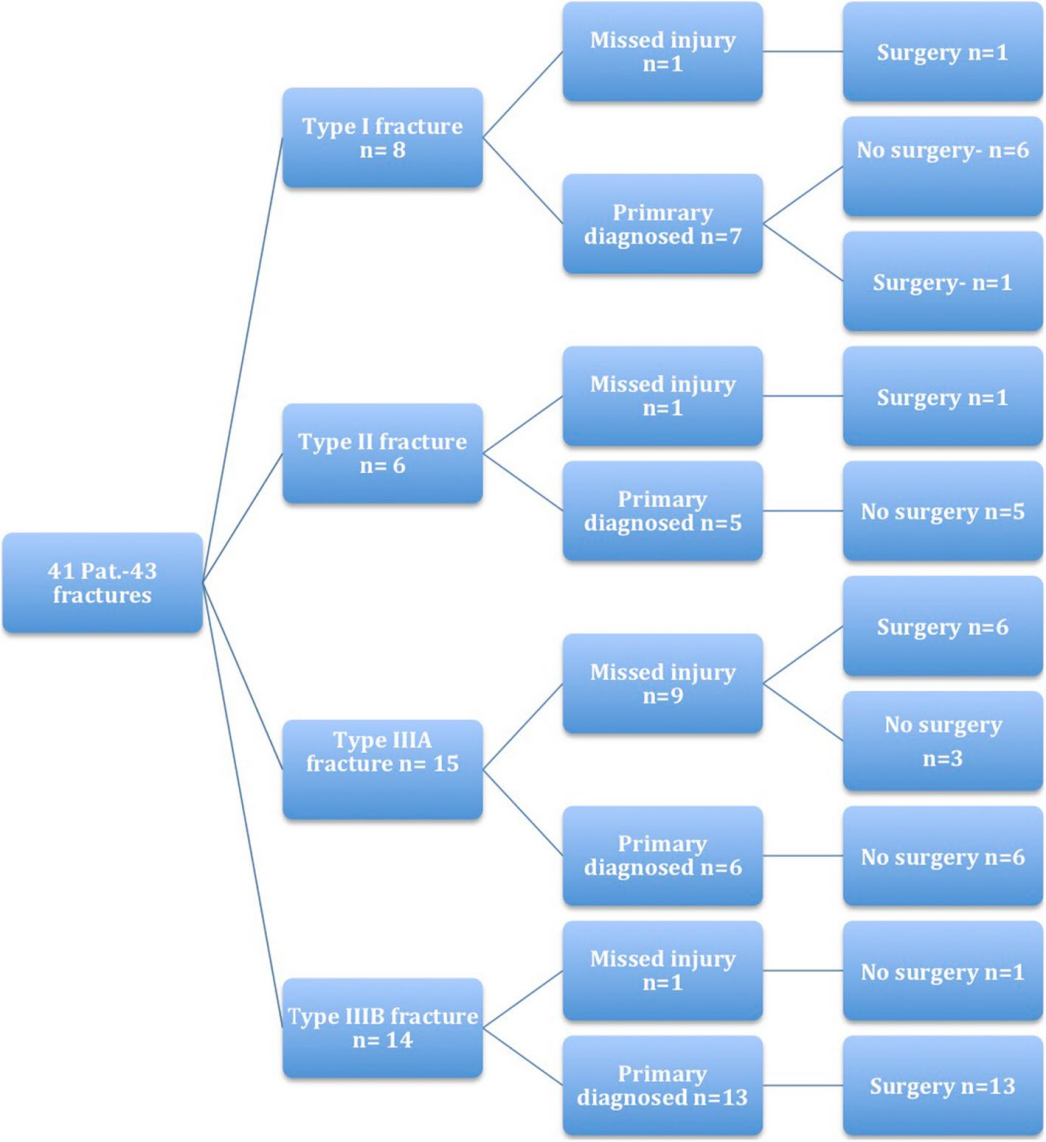
Schematic presentation of the fracture types and their treatment

Based on the existing Degan classification, fractures were classified in all patients. However, this was done using sagittal CT scans. The original type III was further subdivided into subtype A (intraarticularly - not dislocated - without joint step) and subtype B (intraarticularly – dislocated - with joint step) (Fig. 1). The joint step was determined as ≥2 mm. The fragment size was measured using sagittal CT scans with an estimated digital measurement tool (Impax, Agfa, Germany) (Fig. 2). The classification of all fractures and the measurement of the fragment size were carried out by two independent investigators (orthopedic surgeons). In the event of a difference, an agreement was reached in a discussion. Subsequently, the respective treatment method and the further course were documented and evaluated for each classified fracture. Figure 3 gives an overview.

## Results

### Patient selection and demographics

The average FU was 35,5 ± 38,9 months (range 1,5–152 months). In 12 patients (29,3%), the fracture was missed initially, and the patients were referred to our institution because of persistent pain. Twenty-nine patients (70.7%) presented with concomitant injuries. The most common cause of fractures was a twisting injury (*n* = 16, 39%) (Table 1).

**Table 1:** Study Group *n* = 41

Study group	
**Age (years)**	43 ± 13,2 (range 19–79)
**Sex**	
Male	24 (58,5%)
Female	17 (41,5%)
**Initially diagnosed injuries**	29 (70,7%)
**Missed injuries**	12 (29,3%)
**Accident mechanism**	
Twisting injury	16 (39,0%)
Rollover/traffic accident	14 (34,1%)
Fall from a height of over 1,5 m	5 (12,2%)
Fall at ground level	3 (7,3%)
Crush injury	3 (7,3%)
**Isolated fracture**	12 (29,3%)
**Concomitant injuries in 29 patients**	(70,7%)
Talus	8
Os naviculare	8
Metatarsale	8
Os cuboideum	6
Calcaneus neck fracture with the involvement of the CC joint	3
Avulsion CC	2
Avulsion TN	1
Lisfranc dislocation	1
Chopart dislocation	1
Other fractures	10

*CC* Calcaneocuboidal, *TN* Talonavicular

### Fracture classification

We identified eight type I (small and nondisplaced extraarticular avulsion fracture) (18,6%), six type II (displaced extraarticular fracture) (14%), 15 type IIIA (34,9%) and 14 type IIIB (32,6%) fractures. Twelve patients had an isolated APC fracture, 16 patients showed further bony injuries in the area of the Chopart joint, and 13 patients showed additional other lower extremity injuries outside the area of the Chopart joint (Fig. 3, Table 1). The average fragment size measured on the sagittal CT slices in type I and II fractures was 3 × 3 mm and 3 × 3,5 mm, respectively. Type IIIA fractures had an average size of 10 × 10 mm, and type IIIB fractures were on average 13 × 19 mm.

### Type I fractures *n* = 8 (18,6%) (Table 2)

Seven type I fractures were initially diagnosed, five of which were treated nonoperatively (tip toe weight bearing). All of them achieved a return to work after an average of 4,2 months (range 1,5–7). In one patient the fragment was resected with a simultaneous type IIIB fracture (which was treated with ORIF) and also achieved a return to work. Another patient was treated with full weight bearing after resection of an additional lateralis process of tali fracture. He developed osteoarthritis in the CC/TN joint and required TN joint arthrodesis at 34 months.

**Table 2:** Type I fracture *n* = 8 (18,6%)

Case	Concomitant injury	Time from accident to treatment	Treatment	FU in months	Consolidation	Post-traumatic osteoarthritis	Return to work/time
**Initially diagnosed injuries**							
1	Avulsion TN, calcaneus neck fracture	–	Tip toe weight bearing for 6 weeks	92	Yes	No	Yes, after 1,5 months
2	Calcaneus neck + naviculare + talus fracture	–	Tip toe weight bearing for 6 weeks	10	Yes	No	Yes, after 4 months
3	Type IIIA fracture Naviculare + metatarsale 2–4 fracture, soft tissue defect	–	Tip toe weight bearing for 6 weeks	2	Yes	No	Yes, after 7 months
4	Type IIIB Cuboid + big toe joint dislocation fracture	–	Resection (ORIF Type IIIB)	18	Yes	No	Yes, after 3,5 months
5	Talus fracture (Lateralis process of tali)	–	Full load in Aircast	39	Partial	CC (arthrodesis subtalar + TN after 34 months)	Yes, six months after arthrodesis
6	Metatarsale 3–5 fracture	–	Tip toe weight bearing for 12 weeks	34	Yes	Subtalar	Yes, after 5,5 months
7	Talus fracture	–	Tip toe weight bearing for 12 weeks	152	Yes	Subtalar	Yes, after 3 months
**Missed injuries**							
1	–	72 months	CC joint arthrodesis	62	–	CC	Yes, after 3 months

*CC* Calcaneocuboidal, *TN* Talonavicular, *ORIF* Open reduction and internal fixation, *FU* Follow-up

One type I fracture with instability was initially missed and also led to osteoarthritis. The patient had to be treated with CC joint arthrodesis after 72 months.

### Type II fractures *n* = 6 (14%) (Table 3)

The initial diagnosis was made in five patients with type II fractures. All patients were treated nonoperatively (tip toe weight bearing for at least 6 weeks and two patients received an AO fixator due to concomitant injuries). With the exception of one patient, all achieved a return to work (however, one was already retired). Four patients developed posttraumatic osteoarthritis (three in the CC joint, one subtalar and TN joint), however with two patients having an additional cuboid fracture or calcaneus neck fracture.

**Table 3:** Type II fractures *n* = 6 (14%)

Case	Concomitant injury	Time from accident to treatment	Treatment	FU in months	Consolidation	Post-traumatic osteoarthritis	Return to work/time
**Initially diagnosed injuries**							
1	–	–	Tip toe weight bearing for 6 weeks	54	Yes	No	Yes, after 8 months
2	Cuboideum + metatarsale 2–3 fracture, ankle dislocation	–	AO fixator for 6 weeks	2	Pseudarthrosis	Subtalar, TN	No
**3**	Avulsion CC, calcaneus neck fracture, tissue defect	–	Tip toe weight bearing for 6 weeks	120	Yes	CC	Pensioner, walking after 6 weeks
**4**	Naviculare + cuboideum + metatarsale 1–4 fracture, Lisfranc dislocation	–	AO fixator for 6 weeks + 4 weeks tip toe weight bearing	56	Yes	CC	Yes, after retraining
**5**	Os cuneiforme mediale	–	Partial load with an increase of 20 kg every 2 weeks	9	Yes	CC	Yes, after 4 months
**Missed injuries**							
**1**	–	5 months	Open resection	2	–	TN	Yes, after 2 months

*CC* Calcaneocuboidal, *TN* Talonavicular, *ORIF* Open reduction and internal fixation, *FU* Follow-up

One type II fracture was initially missed and resected after five months, but the patient developed osteoarthritis in the TN joint.

### Type IIIA fracture *n* = 15 (34,9%) (Table 4)

A total of six type IIIA fractures could be initially diagnosed. All were treated nonoperatively by tip toe weight bearing/partial load for 6 weeks and five achieved an uncomplicated return to work after an average of 4,6 months (range 1,8–7). One patient had already retired, but was able to walk again after six weeks. However, he developed posttraumatic osteoarthritis in the CC joint.

**Table 4:** Type IIIA fractures *n* = 15 (34,9%)

Case	Concomitant injury	Time from accident to treatment	Treatment	FU in months	Consolidation	Posttraumatic osteoarthritis	Return to work/time
**Initially diagnosed injuries**							
1	Pilon tibiale fracture	–	Tip toe weight bearing for 6 weeks	14	Yes	CC	Pensioner, walking after 6 weeks
2	Metatarsale fracture 1–3	–	Tip toe weight bearing for 6 weeks	2	Yes	No	Yes, after 1,8 months
3	–	–	Tip toe weight bearing for 6 weeks	6	Yes	No	Yes, after 7 months
4	Bone bruise naviculare	–	Partial load with an increase of 20 kg every 2 weeks	15	Yes	No	Yes, after 4,5 months
5	Type I fracture, Naviculare + metatarsale 2–4 fracture, soft tissue defect	–	Tip toe weight bearing for 6 weeks	2	Yes	No	Yes, after 7 months
6	Naviculare fracture	–	Partial load with an increase of 20 kg every 2 weeks	107	Yes	No	Yes, after 3 months
**Missed injuries**							
1	Posterior process of tali fracture	1) 3 months, 2) 26 months	1) Open resection2) CC joint arthrodesis	45	–	CC	No
2	Metatarsale 4 fracture	3 months	ORIF + bone graft	4	Yes	No	Yes, after 4 months
3	Bone bruise talus	1 months	3 weeks of relief, then partial load for 3 weeks	8	Partial	CC	No
4	–	5 months	Open resection	1,5	–	No	Could not be determined
5	–	7 months	Open resection	1,5	–	No	Yes, after 14 days
6	–	32 months	Open resection	78	–	No	Yes, after 14 days
7	–	3,5 months	Tip toe weight bearing for 4 weeks	86	Yes	No	Yes, after 1.5 years
8	–	3 months	Full load in a boot	25	Yes	CC	Yes (never stopped)
9	Cuboideum fracture	10 months	ORIF + bone graft	6	Partial	CC	No

*CC* Calcaneocuboidal, *TN* Talonavicular, *ORIF* Open reduction and internal fixation, *FU* Follow-up

A total of nine type IIIA fractures were initially missed and were treated after an average of 7,5 months (range 1–32). Three of these fractures were surgically resected after five, seven and 32 months because of persistent pain. Two of the patients were able to return to work 14 days after surgery, and the third was lost to further follow-up. Another patient received a resection of the fracture fragment 3 months post-trauma, and at 26 months post-trauma, he required arthrodesis of the CC joint (no return to work during FU of 45 months).

Two other patients received an ORIF + bone graft three and 10 months after trauma and subsequent diagnosis. Return to work was achieved in only one of those patients in the period of the FU of four and 6 months. The other developed posttraumatic osteoarthritis in the CC joint.

Another patient with a fracture that was initially missed and diagnosed after 3 months, who continued to be treated nonoperatively under full weight bearing after the diagnosis, developed posttraumatic arthrosis in the CC joint.

Two other patients were treated with tip toe weight bearing/partial load after the diagnosis was made; only one was able to return to work after 1,5 years. The other developed posttraumatic osteoarthritis in the CC joint.

### Type IIIB fracture *n* = 14 (32,6%) (Table 5)

Patients with an initially diagnosed type IIIB fracture received surgical treatment. 11 patients were treated with ORIF (plate), one received CC joint arthrodesis and one had the fragment resected. In addition, three patients received a temporary bridging of the CC joint. A return to work was achieved in all of these patients after an average of 9,6 months (range 3,5–24), and two patients had already retired. In one patient, however, pseudarthrosis developed without further treatment.

**Table 5:** Type IIIB fractures *n* = 14 (32,6%)

Case	Concomitant injury	Time from accident to treatment	Treatment	FU in months	Consolidation	Posttraumatic osteoarthritis	Return to work/time
**Initially diagnosed injuries**							
1	Above knee amputation opposite leg	–	ORIF (plate)	50	Yes	No	Yes, after 24 months
2	Talus fracture (Lateralis process of tali)	–	ORIF (plate) + temporary arthrodesis (bridging plate)	12	Yes	CC + subtalar	Yes, after 12 months began retraining
3	Tibia + talus + malleolus med fracture	–	ORIF (plate)	92	Pseudarthrosis	CC + subtalar	Yes, after 17 months
4	Talus fracture	–	ORIF (plate)	7	Yes	No	Yes, after 7 months
5	Type I fracture, Cuboid + big toe joint dislocation fracture	–	Resection type I + ORIF (plate)	18	Yes	No	Yes, after 3,5 months
6	Os naviculare + talus fracture	–	ORIF (plate)	8	Yes	No	Yes, after 8 months
7	Os naviculare fracture	–	ORIF (plate)	97	Yes	Subtalar	Yes, after 10 months
8	Fibula + Os naviculare fracture	–	ORIF + temporary arthrodesis (bridging plate)	6	Yes	Subtalar	Pensioner, walking after 5 months
9	Chopart dislocation, naviculare+ cuboideum fracture, metatarsale fractures	–	Arthrodesis CC joint	9	Yes	No	Yes, after 10 months
10	Cuboideum fracture	–	ORIF (plate)	39	Yes	CC	Yes, after 4 months
11	Chopart dislocation, naviculare + cuboideum fracture	–	Resection+temporary arthrodesis	5	–	Subtalar	Yes, after 7 months
12	–	–	ORIF (plate)	14	Yes	No	Yes, after 3 months
13	–	–	ORIF (plate)	49	Yes	CC	Pensioner, walking after 5 months
**Missed injuries**							
1	–	1.5 months	Full load in a boot	20	No X-ray	?	Pensioner

*CC* Calcaneocuboidal, *TN* Talonavicular, *ORIF* Open reduction and internal fixation, *FU* Follow-up

Posttraumatic osteoarthritis in the CC joint was found in four patients; (one with additional cuboideum fracture).

### Complications

Overall, patients with delayed diagnosed APC fractures showed a significantly higher rate of complications [*X*^2^ (1, *N* = 43) 18,348, *p* < 0.000] and required surgical treatment more often than patients with initially diagnosed fractures [*X*^2^ (1, *N* = 43) 6.779, *p* < 0.009].

## Discussion

The aim of this study was to investigate the fracture of the APC, which is considered in only a few studies in the literature, proposing a modification to the conventional Degan classification to better identify the underlying fracture entities [5, 10–14, 16, 17, 21, 22], Utilizing this modified Degan classification, the treatment methods and clinical and radiological results of the fracture were examined to specify treatment algorithms and recommendations. To the best of our knowledge, this is one of the largest patients population described along with a study by Hellpap from 1962 (*n* = 47) [22].

The literature on APC fractures is very inconsistent with mainly case reports (initially diagnosed as well as overlooked fractures) with different treatment methods described, such as a nonoperative procedure, ORIF, open and arthroscopic resection of the fracture fragment or freshening of the nonunion site [10–14, 16, 17, 21]. Studies with high numbers of patients are rare and often without a uniform classification [1, 2, 5, 6, 8, 15, 22, 23]. In 1982, Degan et al. described one of the largest case series with 25 patients. Although he developed a classification of the fracture, a precise classification of all 25 patients and a uniform therapy based on this classification is missing. Eighteen of these 25 patients were treated conservatively using cast immobilization (between 2 and 10 weeks), which was described as successful. In the other seven patients, the fragment was resected; however, patients with initially overlooked fractures were also included. It was reported that five of the seven resected fractures were type III fractures, the others were not classified [5]. A clear therapy recommendation based on the classification is therefore not given.

To be able to give a recommendation based on a uniform classification, we used this classification created by Degan, which was created on the basis of lateral X-ray images [5]. Since all 41 patients of our study had an extended diagnosis in the sense of CT, all fractures were classified retrospectively using the sagittal CT slices. In type III fractures, the improved diagnostics allowed for further differentiation: injuries with noticeable disruption of the joint line (joint step) and undislocated type III injuries. The latter were therefore more often overlooked in X-ray imaging, which significantly impacted the further course of treatment. For this reason, we considered that an expansion of the classification into types IIIA and B would allow a more differentiated analysis. Based on this subdivision, it was shown that good results could be achieved with a nonoperative method (in the sense of tip toe weight bearing or partial load in a boot for 6 weeks) for initially diagnosed type IIIA fractures, regardless of further injuries. Here all patients achieved a return to work. Type III B fractures that were initially diagnosed were all treated surgically in this study because of a large fragment with a joint step. 92% patients showed a consolidation, and achieved a return to work. Surgical treatment thus achieved good results. However, as shown in one patient, a risk of pseudarthrosis also exists with this type of fracture. In the case of type I and II fractures, we were also able to show that a conservative procedure (with the limitations mentioned above) leads to good results. Apart from one, all patients achieved a return to work.

In comparison, the patients with initially missed injuries showed significantly poorer results and significantly higher rate of complications. 66.6% missed APC injuries were secondarily treated surgically because of symptoms, 75% of which were type IIIA fractures. Posttraumatic osteoarthritis was found in 50% of the patients; two patients required arthrodesis of the CC joint, and three did not achieve a return to work during the period of the FU. A better outcome might have been achieved with an immediate diagnosis using CT/MRT and the therapy along our proposed guidelines according to the modified classification.

In a study by Dhinsa et al. (2019), a therapy algorithm was created based on a literary review [9]. This algorithm states that nondisplaced and minimally displaced small fractures can be treated nonoperatively with nonweight bearing in a boot for 6 weeks, and large type III fractures should be treated surgically. Initially overlooked fractures are described to have a negative impact on the outcome. These results largely agree with the results of this study, but in our study, it was also possible (using the extended classification) to successfully treat larger type IIIA (nondisplaced) fractures with an average size of 10 × 10 mm (in the sagittal layers) nonoperatively.

In another recent study from 2019, Massen et al. described a different approach with a purely conservative procedure with full weight bearing in all types of APC fractures. Of the 27 patients with follow-up, 48% were classified as having type I fractures, 33% were classified as having type II fractures, and 19% were classified as having type III fractures. However, all injuries other than those to the Chopart joint were excluded here, and no statement was made about possible instability and posttraumatic osteoarthritis (but with an average Karlsson score of 90 with a maximum of 100 points) [2]. In contrast, our study showed an increased incidence of type III fractures (type I 18.6%, type II 14%, type IIIA 34.9% and type IIIB 32.6%). Nevertheless, these results show that with APC fractures without further injuries outside the Chopart joint, in addition to the six-week rolling load/partial weight bearing in a boot, full weight bearing appears to be possible and should be further discussed and examined. Due to the additional fractures that usually existed in our patient population, this would often not have been possible. Furthermore, especially in the case of type IIIA fractures, we consider immobilization to be the safe choice of therapy due to the involvement of the joint. We make this argument on the basis of the overall rate of posttraumatic osteoarthritis after type IIIA fractures; the complicated, lengthy processes after overlooked injuries observed in our study as well as the cases described in the literature; and the reported long course of injury even in the case of initially recognized injuries [5, 9, 11, 24].

In addition, a study by Hirschmann et al. showed that with APC fracture, further injuries of the Chopart line were present in 76% of fractures on MRI [7]. In our study, concomitant injuries were also found with 62% of the initially diagnosed fractures, although only two patients initially received an MRI. Andermahr et al. as well as other studies described and classified the possibility of additional ligamentous injuries in the area of the CC joint with the risk of permanent disability and impaired function in the case of missed injuries [25–28]. The risk of overlooking an additional ligamentous injury is therefore still present, which is why we would advise against immediate full weight bearing to avoid chronic consequential damage. Further studies are necessary to investigate when the entire Chopart joint is stable to avoid immobilization in type I to type II fractures.

### Limitations

This study has some limitations. Due to the frequent additional injuries, the time period to return to work cannot be related to only the fracture of the APC. Furthermore, conservative management was not always been uniform, and there was a lack of objective outcome scores, which, due to the other injuries, could not be generated purely in relation to the APC injury. Some patients had only a brief follow-up. Often, the addresses of these patients could no longer be determined, or the patients did not want a further examination if they were free of symptoms. There was also no corresponding control group for the therapy of all different groups. Furthermore, we cannot make any statements about the number of possible asymptomatic and thus never diagnosed missed injuries.

## Conclusion

The modified Degan classification used here was proven useful and is now used in our clinical setting to aid in the decision-making of treatment options. Tip toe weightbearing in a boot for 6 weeks for type I to type IIIA injuries and surgical treatment of type IIIB fractures using ORIF showed good results. To avoid the poor outcome of an overlooked injury, especially for type IIIA fractures, CT or MRI should be performed if clinically suspected.

## Data Availability

The datasets used and/or analysed during the current study are available from the corresponding author on reasonable request.

## Abbreviations

APC: Anterior process of the calcaneus
CT: Computed tomography
CC: Calcaneocuboidal
FU: Follow-up
MRI: Magnetic resonance imaging
ORIF: Open reduction and internal fixation
TN: Talonavicular
